# Variation in postoperative non-steroidal anti-inflammatory analgesic use after colorectal surgery: a database analysis

**DOI:** 10.1186/1471-2253-14-18

**Published:** 2014-03-20

**Authors:** Hans-Christian Pommergaard, Mads Klein, Jakob Burcharth, Jacob Rosenberg, Jørgen B Dahl

**Affiliations:** 1 Department of Surgery, Herlev Hospital, University of Copenhagen, Herlev Ringvej 75, DK-2730 Herlev, Denmark; 2 The Scandinavian Postoperative Pain Alliance (SCAPALLI), Department of Anaesthesia, Centre of Head and Orthopaedics, Rigshospitalet, University of Copenhagen, Blegdamsvej 9, 2300 Copenhagen Ø, Denmark; 3 Hindegade 5, 4. tv., 1303 Copenhagen K, Denmark

**Keywords:** Postoperative analgesia, Non-steroid anti-inflammatory drugs, NSAIDs, Multimodal analgesia, Pain treatment

## Abstract

**Background:**

Non-steroid anti-inflammatory drugs (NSAIDs) have been proposed as part of a multimodal postoperative analgesia in patients operated for colorectal cancer. However, whether these drugs are prescribed and taken by the patients have not been evaluated. The aim of this study was to quantify the postoperative use of NSAIDs in these patients.

**Methods:**

Data from patients operated for colorectal cancer between January 1, 2006 and December 31, 2009 were collected from the Danish Colorectal Cancer Group’s (DCCG) prospective database. From the electronically registered medical records, data for the use of the two NSAIDs diclofenac and ibuprofen were recorded. The data from six colorectal departments in eastern Denmark were compared.

**Results:**

Of the 2,754 patients analyzed overall, 40.6% received NSAIDs as part of their analgesic treatment. The percentage of the patients receiving NSAIDs, receiving a pre-defined dosage as a minimum and receiving NSAIDs as p.r.n. medication, and the type of NSAID were significantly different both between department and within departments. The median dose of ibuprofen and diclofenac were 1200 mg (400–2,400 mg) and 100 mg (50–200 mg), respectively.

**Conclusions:**

The large variation between and within the departments points to an inconsistency in the use of multimodal post-operative pain treatments. This may be a result of insufficient evidence on procedure specific pain treatments and possibly a lack of compliance to existing guidelines. High-quality large-scale studies are warranted to form the basis for guidelines for postoperative analgesic treatment.

## Background

In spite of much international focus on postoperative pain treatment, 86–89% of patients still experience moderate to severe pain after surgery [1,2]. Hence, postoperative pain management requires optimization. A solution may be the use of multimodal postoperative analgesia, which reduces opioid related side effects [3-5]. As part of such treatment, non-steroid anti-inflammatory drugs (NSAIDs) are often advocated as an important component [3]. However, surgeons may be reluctant to use NSAID due to concerns about side effects. Our hypothesis was that NSAIDs may not be prescribed or actually taken by the patients as part of a multimodal post-operative analgesic regimen. This has not been evaluated for colorectal surgery and it remains unclear whether consensus exists between departments regarding this treatment.

The aim of this study was to evaluate the type, dosage and extent of NSAIDs used at six major colorectal surgery centers in eastern Denmark, together comprising 39% of the total number of operations performed in the country [6].

## Methods

Data from patients operated for colorectal cancer between January 1, 2006 and December 31, 2009 were collected from the Danish Colorectal Cancer Group’s (DCCG) prospective database, which has a national data completeness rate of 99% [7]. To evaluate the analgesics prescribed to these patients, electronically registered medical records were assessed. In Denmark, all medical drug prescriptions for patients in hospitals utilize a computer-based medical prescription system, where all administered drugs are documented. Hence, a dosage of a drug cannot be administered to a patient without electronic registration.

Data from prescribed postoperative analgesics were used for patients operated at six major colorectal surgery centers in eastern Denmark. All centers had specific local guidelines for postoperative analgesic treatment. The same cohort used in this study has been described previously [8], however with a different focus and data analyses. Patients were included if having available electronic medical records and if having received an elective operation between January 1, 2006 and December 31, 2009 for either colonic or rectal cancer with a primary anastomosis.

A pre-study evaluation found that 0.4% of the patients in this study population received other NSAIDs than ibuprofen and diclofenac. Thus, we chose to focus the study on these two drugs as part of the multimodal postoperative pain treatment. Hence, other opioid sparing drugs, such as alternative NSAIDs or paracetamol, were not evaluated. Data for the postoperative NSAID consumption were collected from electronic medical records, which were registered for each patient only when the medication was prescribed and actually taken by the patient. The following variables were registered:

•Use of the NSAIDs ibuprofen or diclofenac as part of the postoperative analgesic regimen (yes/no).

•The type of NSAID

•Whether the patients receiving NSAID, received a pre-defined dose of the specific drug as a minimum. For ibuprofen, this dosage was defined as 800 mg/day for at least two days and for diclofenac, the dosage use was defined as 50 mg/day for at least two days.

•NSAIDs used as p.r.n.

Patients’ age and dosage of NSAIDs were reported as median (range), since these data were not normally distributed. Remaining variables were reported as percentages. Chi-square test was used to evaluate the distribution of dichotomous outcomes between departments, whereas Kruskal-Wallis test was used to compare dosages of NSAIDs between departments. A p-value less than 0.05 was regarded statistically significant. SPSS version 19 (SPSS Inc, Chicago, Illinois, USA) was used for statistical analyses.

Approval was obtained from the Danish data protection agency (license number 2008-41-2484). The study was not eligible for ethics committee approval according to Danish law, since we had no contact with patients, did not perform any interventions or used biological material.

## Results

The patients had a median age of 70 years with an equal distribution between genders (Table 1).

**Table 1:** Demographic data for the included patients


Age, median (range)	70 (22–97)
Gender (males/females)	52.3%/47.7%
ASA-class	23.4% (1), 60.2% (2), 15.7% (3), 0.7% (4)

ASA: American Society of Anesthesiologists.

**Figure 1:**
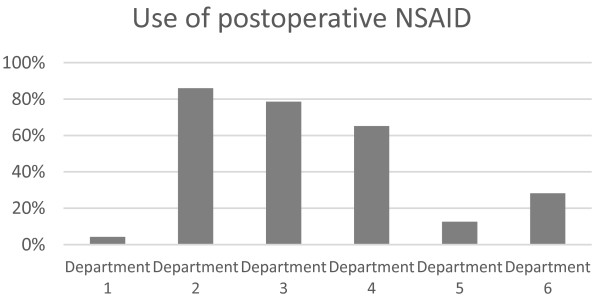
The percentages at the different departments using NSAIDs as postoperative pain treatment.

Of the 2,754 patients, 40.6% (1,117) received NSAIDs as part of their postoperative analgesic treatment. When evaluated on department level, there was a wide variation between departments in the use of NSAIDs (p < 0.001, chi-square test), (Figure 1). Departments 2–4 used NSAIDs frequently, whereas the remaining departments rarely used NSAIDs.

**Figure 2:**
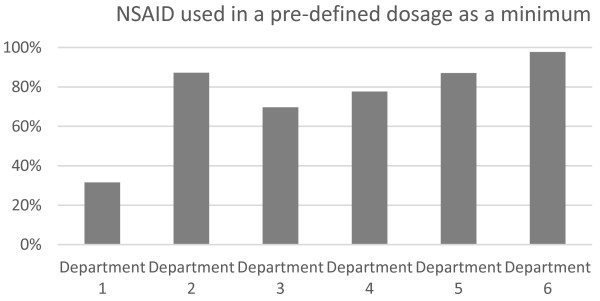
The percentages at the different departments using NSAIDs in a pre-defined dosage as a minimum.

The percentage of the patients receiving NSAIDs who received the pre-defined dosage as a minimum was also different between the departments (p < 0.001, chi-square test), (Figure 2). For department 1 and partly for department 3, NSAIDs were generally used below this dosage. However, in the remaining departments the majority of patients received the pre-defined dosage of NSAIDs.

The type of NSAID used differed between departments (p < 0.001, chi-square test). However, most departments used ibuprofen in the majority of the patients except for department 6, in which diclofenac was used in more than 80% of the patients (Tables 2 and 3).

**Table 2:** Median and range for ibuprofen doses used in the different departments

**Department**	**1**	**2**	**3**	**4**	**5**	**6**
Number of patients	79	5	296	203	40	30
Percentage of total NSAID use	83,3%	77,5%	96,1%	99,0%	88,9%	14,1%
Dosage (median)	1,200	1,600	1,200	1,600	1,200	1,200
Range	400–1,800	1,200–1,800	400–1,800	800–2,400	800–1,800	600–1,800

**Table 3:** Median and range for diclofenac doses used in the different departments

**Department**	**1**	**2**	**3**	**4**	**5**	**6**
Number of patients	23	1	12	2	5	183
Percentage of total NSAID use	16,7%	22,5%	3,9%	1,0%	11,1%	85,9%
Dosage (median)	150	50	100	125	150	100
Range	150–200	NA	50–150	100–150	100–200	75–200

NA: not applicable.

Of the patients receiving the pre-defined dosage of NSAIDs as a minimum, the specific daily dosage differed between the departments (p = 0.025, Kruskal-wallis test), with a median dosage of 1200 mg (400–2,400 mg) for ibuprofen for all departments (Table 2). For diclofenac, the median dosage was 100 mg (50–200 mg), with a statistically significant difference between departments (p < 0.001, Kruskal-wallis test), (Table 3).

**Figure 3:**
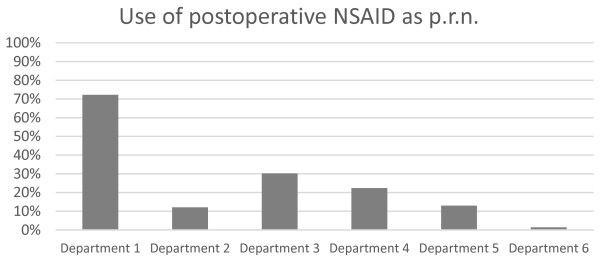
The percentages at the different departments using NSAIDs as p.r.n. in their postoperative pain treatment.

Of the patients receiving NSAIDs, departments differed in whether NSAIDs were used as p.r.n. medication in contrast to regular medication (p < 0.001, chi-square test), (Figure 3). Department 1 used NSAIDs as p.r.n. medication frequently. However, this use was less pronounced in the other departments.

## Discussion

This study found that use of NSAIDs as a part of the postoperative analgesic regimen differed widely both between and within colorectal surgery departments in eastern Denmark. Moreover, the type of NSAID differed between the departments. However, ibuprofen was the primary NSAID used.

The variation in the use of NSAIDs, the dosage and the type between departments may reflect conflicting guidelines in the different departments. Thus, there is obviously a lack of consensus on the proper postoperative analgesic regimen to use, at least concerning NSAIDs. Procedure specific regimens for patients operated with colorectal resection have been proposed [9,10]. Such guidelines should be based on the best available evidence and whether they are implemented into clinical practice may depend on the quality of that evidence and the dissemination of knowledge to the clinicians.

Even though each department in the study had local guidelines, a variation in the use of NSAIDs was observed within the departments. This may be a result of the clinicians not fully adhering to the guidelines, possibly due to concern of adverse effects, or that the clinicians were not completely informed about these. In Denmark, pre-specified analgesic regimens may be available in the electronic medical systems. However, it has been shown that actual instructions in form of written procedure specific guidelines seldom exist in departments [5]. The range of the prescribed doses shows that some of departments administrated quite low doses (400 mg for Ibuprofen) of NSAID to the patients. If the administration of such a low dosage reflects what was prescribed, this may illustrate a lack of knowledge about proper dosage of NSIAD among clinicians and therefore education may be needed. However, the variation may also be explained by discrepancies between the medication prescribed and the medication actually given to the patients. In situations where the patient is not in pain, patients or caregiver may think that the prescribed medication may not be necessary. However, there are good reasons to give analgesics even during a period without pain in order to prevent break-through pain [11].

One of the main reasons to use multimodal analgesic post-operative treatment is to reduce opioid consumption and thereby limit opioid induced nausea and other side-effects [3-5]. Despite these obvious benefits, studies have shown that patients do not receive multimodal pain treatment to a sufficient degree [2,5,12].

NSAIDs are commonly used as part of a multimodal analgesic regimen [10,13]. However, it has been questioned whether NSAIDs may have detrimental effects for patients receiving colorectal anastomoses. Despite this, such regimens are recommended after colorectal surgery [9,10,13], but no consensus exists on which type of NSAID to use. In some guidelines, COX-2 selective drugs have been recommended [9,10]. Nevertheless, other researchers have banned NSAIDs, such as diclofenac and celecoxib, due to a possibly increased risk of anastomotic leakage in the patients in addition to other potential complications [8,14]. Several studies have shown that these NSAIDs may increase the risk of anastomotic leakage [8,15,16]. Thus, there is increasing evidence that these drugs should not be used for patients receiving colorectal anastomoses. A recent study suggests that non-selective NSAIDs may be more harmful than COX-2 selective NSAIDs with regards to anastomotic leakage [17]. In the present study, diclofenac was used in a proportion of the patients. However, due to the recent increased awareness of the negative effects of this drug, the use of diclofenac may have decreased after the time period investigated in this study.

The strengths of this study were 100% data completeness from the electronic medical records and a precise knowledge on whether a prescribed drug was actually taken by the patients. However, we only evaluated ibuprofen and diclofenac, and other opioid sparing drugs, such as paracetamol, were not evaluated in this study. We evaluated the daily dose of the NSAIDs and whether the drug was given for more or less than two days. However, the precise number of days was not recorded.

As described earlier, procedure specific guidelines already exist for colorectal surgery [10,13]. However, quality of the evidence for many specific procedures, including colorectal surgery, is still not sufficient. The evidence for an additive or synergistic effect of various drug combinations is sparse, as is the evidence for particular dosages. A basic recipe for post-operative analgesia should be defined based on evidence from large-scale studies concerning safety and efficacy.

## Conclusion

In conclusion, large variations in the use of postoperative NSAIDs exist both between and within departments. This points to an inconsistency in the use of multimodal postoperative pain treatments and a lack of consensus on the treatment regimens, and may be a result of insufficient evidence on procedure specific pain treatments. Thus, large-scale studies are needed to produce guidelines for post-operative analgesic treatment based on high-quality evidence.

## Competing interests

The authors declare that they have no competing interests.

## Authors’ contributions

HCP, MK, JB, JR and JBD made substantial contributions to the conception and design of the study. HCP and MK made the data analysis. HCP drafted the manuscript. HCP, MK, JB, JR and JBD made critical revisions of the manuscript. All authors read and approved the final version of the manuscript.

## Pre-publication history

The pre-publication history for this paper can be accessed here:

http://www.biomedcentral.com/1471-2253/14/18/prepub

## References

[B1] Apfelbaum JL Chen C Mehta SS Gan TJ Postoperative pain experience: results from a national survey suggest postoperative pain continues to be undermanaged Anesth Analg 2003 97 2 534 540 table of contents 10.1213/01.ANE.0000068822.10113.9E 12873949

[B2] Fletcher D Fermanian C Mardaye A Aegerter P Pain and Regional Anesthesia Committee of the French Anesthesia and Intensive Care Society A patient-based national survey on postoperative pain management in France reveals significant achievements and persistent challenges Pain 2008 137 2 441 451 10.1016/j.pain.2008.02.026 18417292

[B3] Kehlet H Postoperative opioid sparing to hasten recovery: what are the issues? Anesthesiology 2005 102 6 1083 1085 10.1097/00000542-200506000-00004 15915017

[B4] Kehlet H Wilmore DW Evidence-based surgical care and the evolution of fast-track surgery Ann Surg 2008 248 2 189 198 10.1097/SLA.0b013e31817f2c1a 18650627

[B5] Mathiesen O Thomsen BA Kitter B Dahl JB Kehlet H Need for improved treatment of postoperative pain Dan Med J 2012 59 4 A4401 22459715

[B6] Nationwide database for cancer of the colon and rectum - yearly report [http://dccg.dk/03_Publikation/02_arsraport_pdf/aarsrapport_2010.pdf]

[B7] Gogenur I Ingeholm P Iversen LH [Danish colorectal cancer database] Ugeskr Laeger 2012 174 42 2525 23079428

[B8] Klein M Gogenur I Rosenberg J Postoperative use of non-steroidal anti-inflammatory drugs in patients with anastomotic leakage requiring reoperation after colorectal resection: cohort study based on prospective data BMJ 2012 345 e6166 10.1136/bmj.e6166 23015299 PMC3458793

[B9] PROSPECT working group [http://www.postoppain.org/]

[B10] Joshi GP Bonnet F Kehlet H PROSPECT Collaboration Evidence-based postoperative pain management after laparoscopic colorectal surgery Colorectal Dis 2013 15 2 146 155 10.1111/j.1463-1318.2012.03062.x 23350836

[B11] Carr DB Goudas LC Acute pain Lancet 1999 353 9169 2051 2058 10.1016/S0140-6736(99)03313-9 10376632

[B12] Benhamou D Berti M Brodner G De Andres J Draisci G Moreno-Azcoita M Neugebauer EA Schwenk W Torres LM Viel E Postoperative Analgesic THerapy Observational Survey (PATHOS): a practice pattern study in 7 central/southern European countries Pain 2008 136 1–2 134 141 17703887 10.1016/j.pain.2007.06.028

[B13] Holst Andersen LP Werner MU Rosenberg J Gogenur I Procedure specific pain management in relation to laparoscopic colonic surgery Ugeskr Laeger 2013 175 11 721 725 23480883

[B14] Klein M Holst Andersen LP Gogenur I Rosenberg J COX-2 selective NSAIDs should not be used after colorectal surgery Colorectal Dis 2013 15 9 1186 23701434 10.1111/codi.12298

[B15] Holte K Andersen J Jakobsen DH Kehlet H Cyclo-oxygenase 2 inhibitors and the risk of anastomotic leakage after fast-track colonic surgery Br J Surg 2009 96 6 650 654 10.1002/bjs.6598 19434706

[B16] Klein M Andersen LP Harvald T Rosenberg J Gogenur I Increased risk of anastomotic leakage with diclofenac treatment after laparoscopic colorectal surgery Dig Surg 2009 26 1 27 30 10.1159/000193329 19153492

[B17] Gorissen KJ Benning D Berghmans T Snoeijs MG Sosef MN Hulsewe KW Luyer MD Risk of anastomotic leakage with non-steroidal anti-inflammatory drugs in colorectal surgery Br J Surg 2012 99 5 721 727 10.1002/bjs.8691 22318712

